# chemfp - fast and portable fingerprint formats and tools

**DOI:** 10.1186/1758-2946-3-S1-P12

**Published:** 2011-04-19

**Authors:** AP Dalke

**Affiliations:** 1 Andrew Dalke Scientific AB, Göteborg, 413 10, Sweden

## 

Fingerprints are conceptually simple but the abstract sequence of 0 and 1 bits are represented in an astonishing variety of forms. The diversity exists for a very practical sense: it's easier for most researchers to create a simple format than it is to search for or advocate a common standard. Incompatible formats often have no immediate or large negative consequence. The problems are more subtle. Ad hoc formats cannot easily be exchanged with other groups. They lack metadata to help track the provenance of a data set. They do not have existing tools for creating and manipulating records, and the tools which are written are often an order of magnitude slower than what an optimized program can achive.

I have developed two file portable file formats for storing the short and dense fingerprints (order 16 K bits or less, with density > 1%) often seen in cheminformatics. The FPS format is a line-based text format using hex fingerprint encoding. It is designed to be readable and easy to generate and parse. The FPB format is a block-based binary format designed for high-performance operations, including optimized ordering for sublinear Tanimoto searches [1]. The format descriptions are freely available at [2] along with the chemfp Python package to generate, convert, and work with the formats. It includes a C library and extension for fast parsing and fingerprint operations.
